# Caught in the crossfire: biodiversity conservation paradox of sociopolitical conflict

**DOI:** 10.1038/s44185-024-00044-8

**Published:** 2024-04-15

**Authors:** Bona Abigail Hilario-Husain, Krizler Cejuela Tanalgo, Sarrah Jane C. Guerrero, Francisco Gil N. Garcia, Tessie E. Lerios, May Eva Z. Garcia, Renee Jane Alvaro-Ele, Meriam Manampan-Rubio, Sedra A. Murray, Lothy F. Casim, Jamaica L. Delos Reyes, Kier Celestial Dela Cruz, Sumaira S. Abdullah, Shiela Mae Prince Balase, Jeaneth Magelen V. Respicio, Asraf K. Lidasan, Zafrullah S. Buday, Ma. Teodora N. Cabasan, Jonald L. Pimentel, Florie Jane M. Tamon, Angelo Rellama Agduma

**Affiliations:** 1 Ecology and Conservation Research Laboratory (Eco/Con Lab), Department of Biological Sciences, College of Science and Mathematics, University of Southern Mindanao, Kabacan, 9407 Cotabato Philippines; 2 Department of Biological Sciences, College of Science and Mathematics, University of Southern Mindanao, Kabacan, 9407 Cotabato Philippines; 3 Department of Development Communication, College of Arts and Social Sciences, University of Southern Mindanao, Kabacan, 9407 Cotabato Philippines; 4 Department of Agricultural Economics, College of Business, Development Economics and Management, University of Southern Mindanao, Kabacan, 9407 Cotabato Philippines; 5 Molecular Parasitology Research Laboratory, Department of Biological Sciences, College of Science and Mathematics, University of Southern Mindanao, Kabacan, 9407 Cotabato Philippines; 6 Dungguan, Datu Montawal 9610, Maguindanao del Sur, Bangsamoro Autonomous Region in Muslim Mindanao, Marawi, Philippines; 7 Nematology Research Laboratory, Department of Biological Sciences, College of Science and Mathematics, University of Southern Mindanao, Kabacan, 9407 Cotabato Philippines; 8 Department of Mathematics and Statistics, College of Science and Mathematics, University of Southern Mindanao, Kabacan, 9407 Cotabato Philippines; 9 Department of Social Science and Philosophy, College of Arts and Social Sciences, University of Southern Mindanao, Kabacan, 9407 Cotabato Philippines; 10 Guangxi Key Laboratory of Forest Ecology and Conservation, College of Forestry, Guangxi University, Nanning, 530004 Guangxi China; 11 State Key Laboratory for Conservation and Utilization of Subtropical Agrobioresources, Guangxi University, Nanning, 530004 Guangxi China

**Keywords:** Ecology, Behavioural ecology, Conservation biology

## Abstract

The current state of global biodiversity is confronted with escalating threats arising from human-induced environmental changes and a growing array of unpredictable challenges. However, effective conservation efforts are often hindered by limited knowledge, especially in developing economies such as the Philippines. The limitations imposed by these shortfalls in biodiversity knowledge hamper the capacity to protect biodiversity in light of the continuing extinction crisis. Our study revealed that areas with higher conflict levels exhibited lower species richness, fewer occurrence records, and reduced forest cover. This finding provides initial evidence for the relationship between sociopolitical conflict and biodiversity in the Philippines. We posit that the security risks caused by sociopolitical conflicts could have a negative impact on conservation efforts, particularly in terms of monitoring and implementing measures to protect natural resources. The links that bind armed conflict and biodiversity conservation are multifaceted and complex issues that warrant greater scientific and political attention. Finally, we identified 10 meaningful approaches to address shortfalls in biodiversity knowledge in conflicted areas, particularly incorporating conflict-sensitive approaches, considering the geopolitical context and conflict dynamics to adapt and align their strategies with local realities for more effective conservation efforts.

## Introduction

Reducing the rate of global biodiversity loss, halting extinction risks, and preserving intact ecosystems are central to conservation biology^1^. The latest Kunming-Montreal Global Biodiversity Framework aims to safeguard large proportions of species and habitats^2^, and conservation biologists and ecologists rely on accurate and robust biodiversity data to effectively develop conservation priorities^3,4^. Despite the progress made to catalogue biodiversity on Earth^5^, there are persistent gaps in biodiversity knowledge across various areas^6,7^. Consolidating biodiversity information is often challenged by insufficient funding^8^, limited capacity to implement conservation on the ground, and issues related to national security^9^.

Armed conflicts and violent extremism have long been recognised as significant threats to national security, altering social stability^9,10^. The impacts of armed and sociopolitical conflicts on the environment and biodiversity have also gained considerable attention in recent years^10,11^. For instance, the world witnessed the destruction of forests during the Vietnam War^9,12^, the draining of Mesopotamian marshes during the Gulf War^13^, and a decline in wildlife during civil wars in the Democratic Republic of the Congo^14^. At the same time, the 21st-century wars in Afghanistan, Syria, and Ukraine continue to affect biodiversity at an alarming rate^15–18^. Undeniably, armed conflicts and violent extremism can lead to extensive habitat destruction, altering patterns of biodiversity. In fact, of the major armed conflicts between 1950 and 2000, over 90% occurred in countries with biodiversity hotspots, and 80% occurred directly within hotspot areas^10^. The environmental footprints of military activities, explosives, and landmines cause long-lasting damage to ecosystems, leading to the loss of critical habitats for diverse flora and fauna^19^. These activities significantly worsen the problem of biodiversity depletion by promoting unsustainable practices, which disturb delicate ecological equilibriums such as overharvesting species, habitat destruction, and deforestation^20–22^. Moreover, armed conflicts tend to undermine the effectiveness of environmental governance systems, enforce environmental regulations, and safeguard vulnerable ecosystems.

These warfare-driven threats to biodiversity prompted the United Nations (UN) General Assembly in 2001 to declare every 6th of November as the *International Day for Preventing the Exploitation of the Environment in War and Armed Conflict*^23^. Twenty-three years later, many gaps in policy and research still need to be addressed, especially in developing economies, where biodiversity capacity building remains insufficient. Biodiversity hotspots, protected areas, and indigenous territories lack protection under international humanitarian law during armed and sociopolitical conflicts^22^. Previous analyses have demonstrated how sociopolitical conflicts could negatively impact the documentation of biodiversity^10,19^, which consequently limits effective conservation. Yet, there is a lack of well-researched case studies^24^, particularly in the Philippines, a megadiverse country.

Mindanao is the second largest group of islands in the Philippines, consisting of 27 provinces and 33 cities within six administrative regions with an estimated population of 26,252,442 (24% of the country’s population), and its mainland is the seventh most populous island in the world^25^. Its large fertile landmass makes it a major raw material producer in the Philippines, producing around 40% of the country’s agricultural produce and 60% of agricultural exports^26^. In addition, owing to its unique biogeographical history and position, Mindanao is a biodiversity hotspot for diverse flora and fauna^27,28^. Mindanao has over 30 Key Biodiversity Areas (KBAs) that are globally significant sites for biodiversity conservation because of the high concentration of endemic and threatened species, including the globally threatened Philippine Eagle (*Pithecophaga jefferyi*), and other keystone species^28–31^.

**Fig. 1:**
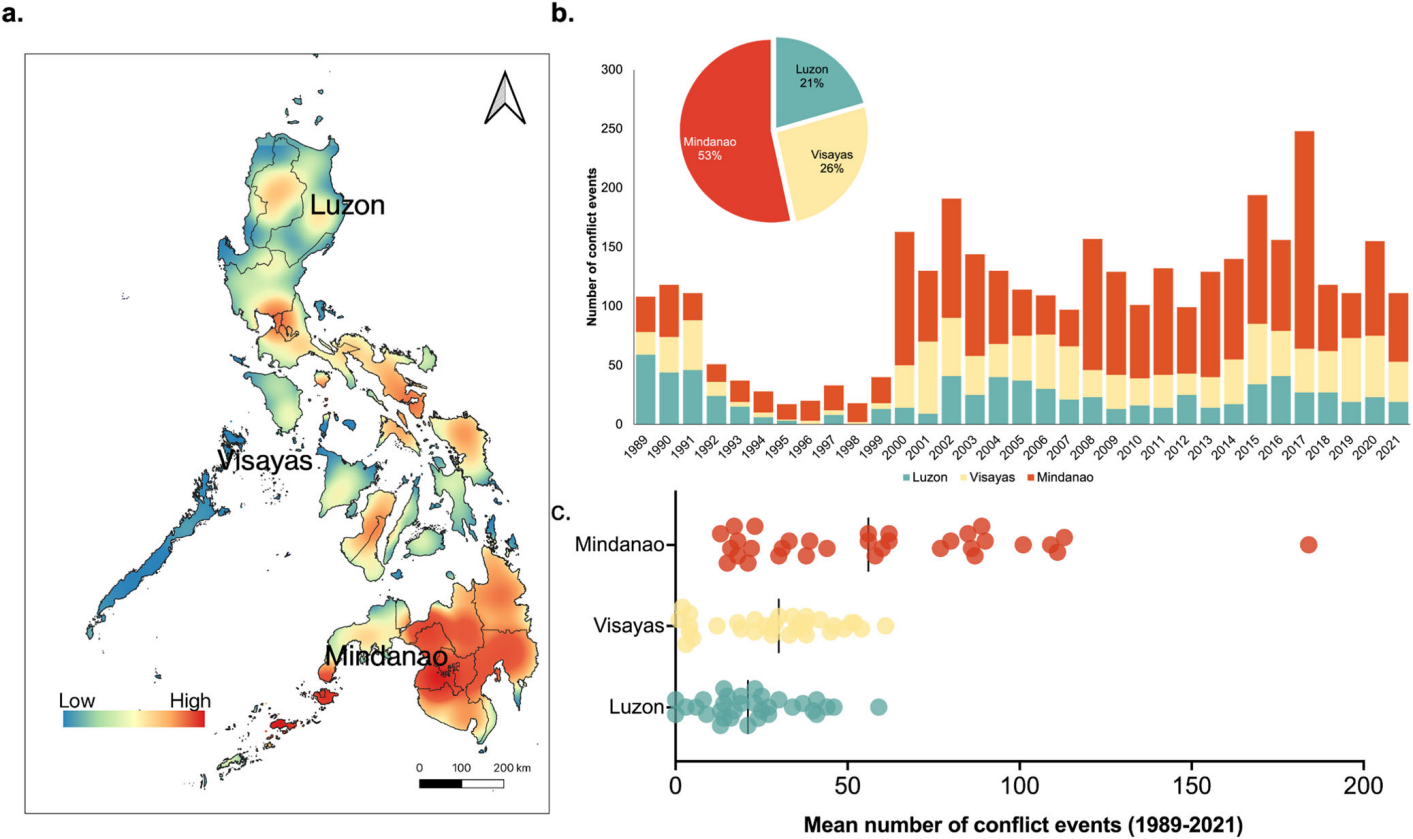
Distribution of war and conflict in the Philippines. **a** Density occurrence, **b** temporal patterns, and **c** average number of conflict events in the Philippines from 1989 to 2021 based on the UN OCHA database^33^.

Whilst Mindanao is known for its rich and diverse wildlife and valuable natural resources, the region confronts a disconcerting reality marked by a confluence of sociopolitical adversities encompassing armed conflicts, religious tensions, feuds among clans, abductions, and other incidents of violence that paint a grim picture of the region^32^. The history of war and armed conflict in Mindanao is complex^32,33^, which can be traced back to the colonial era when the Philippines was under Spanish rule during the 16th century. Spanish colonisers encountered resistance from Muslim communities in Mindanao, who fought against their conversion to Christianity and the imposition of colonial rule^34^. In the 21st century, the Philippine government faced multiple parallel domestic armed conflicts and violence^35^. Data from the United Nations Office for the Coordination of Humanitarian Affairs (OCHA) have estimated that Mindanao has experienced more than half (53%) of the sociopolitical conflicts in the Philippines from 1989 to the present^36^ (Fig. 1).

Contemporary conflicts spanning 60 years in Mindanao might lead to considerable ecological and environmental degradation. Moreover, the collection and analysis of biodiversity information may have been significantly impeded in areas facing sociopolitical and armed conflicts. Apart from direct environmental impacts, sociopolitical conflicts can disrupt research activities, hinder access to remote or affected areas, and create risks for scientists and conservation biologists^20,21,37^. This, in turn, limits our ability to gather accurate data on local flora and fauna, which is essential for making informed decisions about biodiversity conservation and management. Recent Philippine-wide assessments have highlighted differences in survey efforts, notably the low number of studies and recorded species in Mindanao (for example, in bats^38^ and primates^39^). However, the lack of formal studies linking armed conflict to biodiversity knowledge shortfalls in the Philippines is a significant gap.

Addressing the biodiversity knowledge shortfall is crucial for understanding species distribution, the extent of the environmental impacts of conflict, formulation of effective policies and strategies to mitigate these effects, and promoting sustainable development and conservation in the region. Here, we present a perspective highlighting the link between sociopolitical conflicts and biodiversity knowledge shortfalls^6^, specifically in the context of the Southern Philippines. To gain insight into the influence of sociopolitical conflicts on species richness and occurrence records in Mindanao, we analysed the spatial distribution pattern of biodiversity data and remote sensing variables related to habitat transformation (namely tree cover and density, and forest height) within Mindanao and their possible association with sociopolitical conflict events between 2000 and 2021. Finally, we identified meaningful approaches to address biodiversity shortfalls in conflicted areas.

## Results

### Patterns of biodiversity knowledge shortfalls

**Fig. 2:**
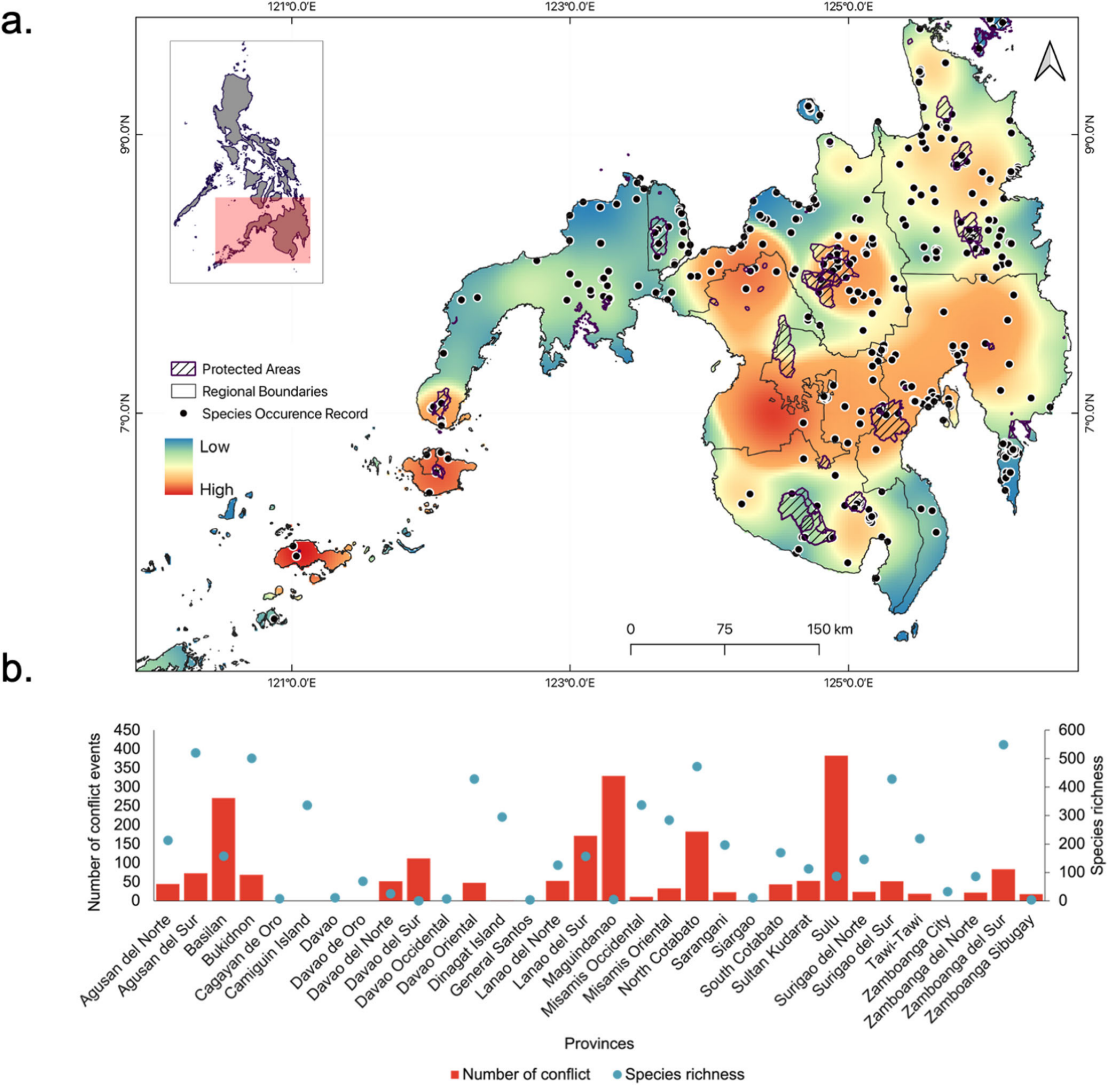
Distribution of war and conflict in Mindanao relative to its provinces. **a** Spatial distribution of species occurrence records (dots) and density of sociopolitical conflict events in Mindanao from 2000 to 2021 and **b** comparison of conflict events and recorded species richness at the provincial level.

**Fig. 3:**
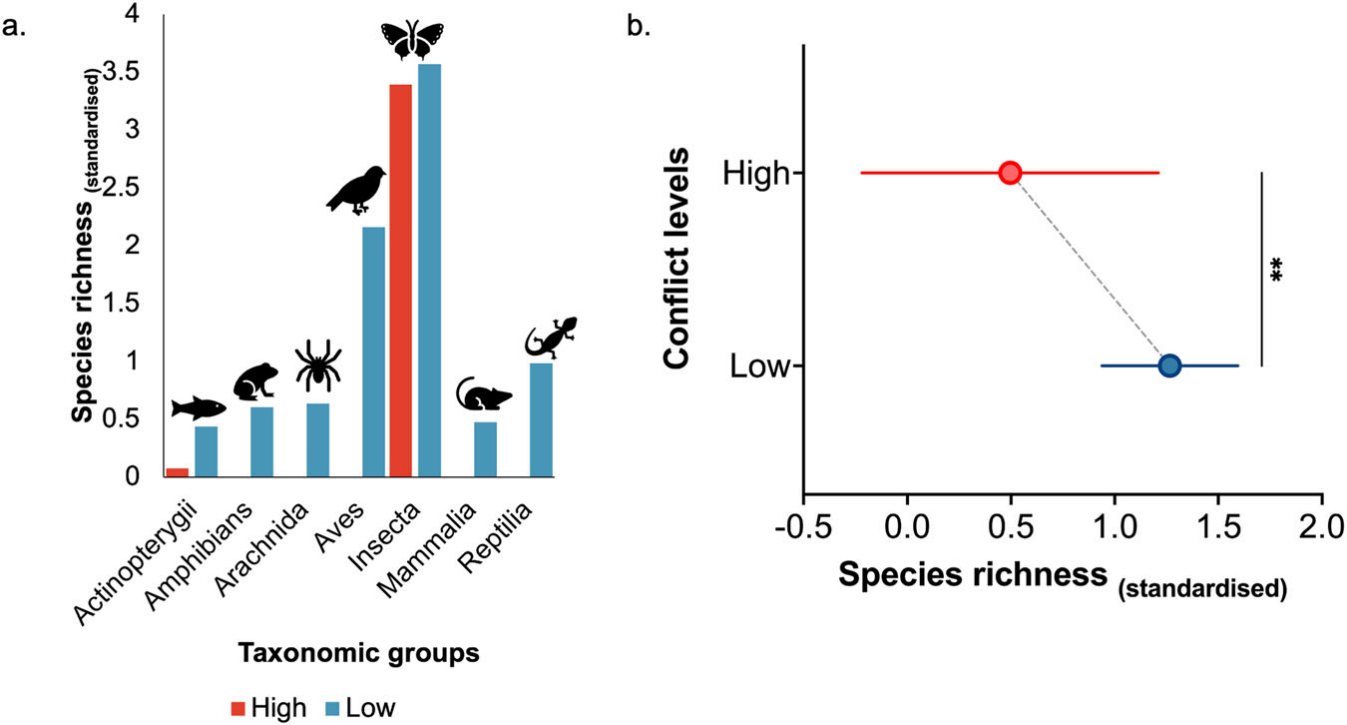
Differences in species richness between low- and high-conflict areas. **a** Comparison of normalised species richness among taxonomic groups and **b** overall group comparison between low- and high-conflict areas. Note: ** indicates significance at *p* < 0.001; whiskers represent 95% CI intervals.

Our analysis revealed how sociopolitical conflict promotes gaps in biodiversity knowledge within a biodiverse island in the Philippines. First, a total of 2174 conflicts (103.52 conflicts/year) were recorded in Mindanao from 2000 to 2021, with the highest levels of violence recorded in Sulu (mean = 82 annually; 18%) and Maguindanao (mean = 329 annually; 15%) provinces (Fig. 2). This has an impact on observed biodiversity. We found a significant difference in observed species richness between high- (mean = 0.50 ± 1.60) and low-conflict (mean = 1.27 ± 2.37) areas (Mann–Whitney *U* test = 1340, *p* = 0.0027) (Fig. 3).

**Fig. 4:**
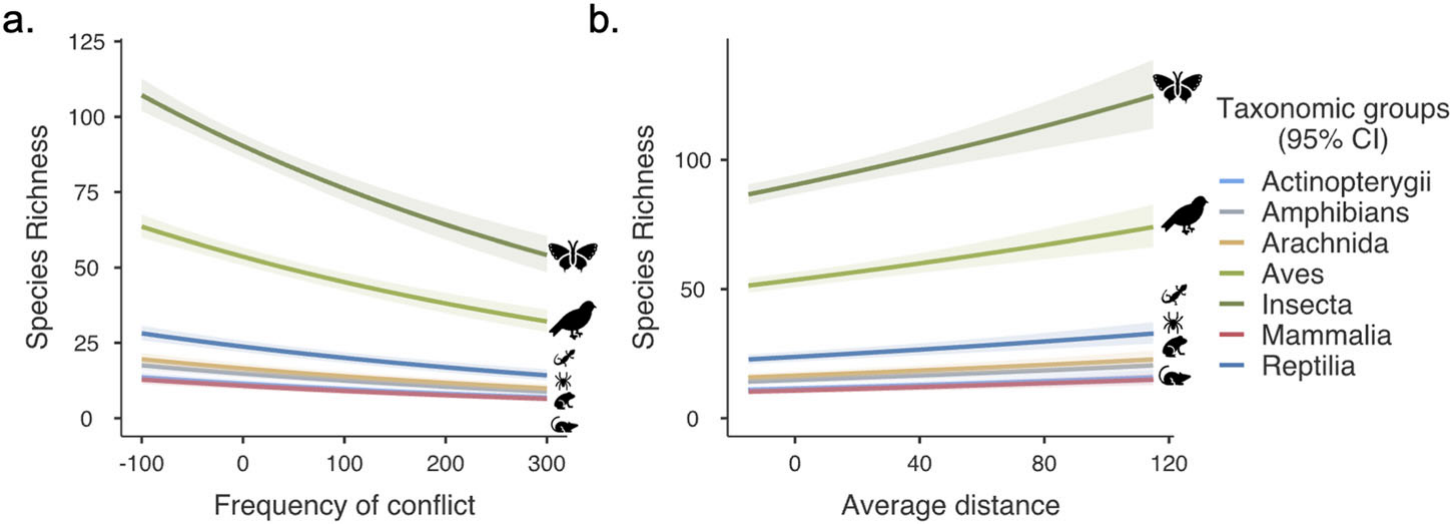
Relationship between conflict and distance from species occurrence. Visualised results of the generalised linear model (Poisson GLM) showing the association between species richness, **a** frequency of events, and **b** average distance at the provincial level. Note: Shading represents the 95% CI. Graphics were from Microsoft Office.

We then modelled the link between species occurrence records and conflict events in Mindanao at the provincial level. The best model for predicting spatial variation in species occurrence records (species record ~1 + conflict events + average distance from conflict events + taxonomic groups, AIC = 7864.53) showed that an increase in the number of conflict events was associated with lower recorded species richness (*β* = –0.002, *p* < 0.0001) (Fig. 4a). Conversely, we showed that the spatial distribution of species richness was higher in areas farther away from conflict events (*β* = 0.003, *p* < 0.0001) (Fig. 4b), particularly for insects (*β* = 2.067, *p* < 0.0001) and birds (*β* = 1.545, *p* < 0.0001).

### Conflict in forests and protected areas

The spatial distribution of conflict events varied across habitat types (*χ*^2^ = 716, df = 6, *p* < 0.0001). The majority of the conflict events fell within open habitats, such as agricultural areas (61%) and grasslands (19%), with only 6% of the recorded conflict events from 2000 to 2021 within protected areas. Moreover, we found a significant negative correlation between a number of fatalities with tree density (Pearson’s *r* = −0.05, *p* = 0.013) and tree cover (%) (Pearson’s *r* = −0.06, *p* = 0.002) but not with canopy height (Pearson’s *r* = −0.04, *p* = 0.054).

## Discussion

Our findings provide valuable insights into the relationship between sociopolitical conflict and shortfalls of biodiversity knowledge in the Philippines. First, our analysis showed that species richness and occurrence decreased in areas with higher numbers and distances to conflict but were taxonomically dependent. Birds and insects exhibited a more robust response to the dependent variables than other taxonomic groups. This can be attributed to their greater detectability, making them easier to sample and collect than other groups like mammals and herptiles^28,31,40^. Unlike these groups, birds and insects are often more conspicuous and exhibit behaviours and characteristics that facilitate easier observation and data collection. This makes them useful indicators of changes in species turnover in conflict zones^24^. Here, we posit that in high-conflict areas, the number of species or their populations have likely declined, potentially resulting in observed biodiversity that falls well below the expected levels due to conflict-related pressures. Another potential reason could be the difficulty in recording and inventorying biodiversity within highly conflicted areas. These areas often require staying in the field, which can be challenging and may deter biologists from making efforts to study biodiversity owing to security risks. Scientists often face challenges when conducting fieldwork in regions that experience ongoing disputes or unstable political situations. These range from personal safety concerns to logistical difficulties in accessing remote and unstable areas. Consequently, it may be impractical to conduct extensive biodiversity assessments in areas marked by high levels of conflict.

Although most areas in Mindanao are now relatively accessible, such as the Ligawasan Marsh in the BARMM region^30^, conducting biodiversity research remains a challenge because of the fear brought about by past conflict events and the disruption of local peace and order in some areas. Several non-state armed groups operate in Mindanao, particularly in Maguindanao, Lanao Del Sur, Basilan, Sulu, and Tawi-Tawi^41^. Other violent tensions, such as clan wars or *‘rido’* and political disputes, brought brutal incidents, such as the Maguindanao massacre in 2011^42^. Another example was the kidnapping of bird watchers in Tawi-Tawi by the *Abu Sayyaf* group in 2012^43^, which prompted foreign and local authorities to advise their citizens, including biodiversity researchers, to refrain from travelling or visiting Mindanao. In regions where security risks impede the ability of scientists to conduct biodiversity assessments, documented biodiversity may underrepresent the actual diversity of species. This disparity can have profound implications for conservation efforts and for our understanding of the true biodiversity status of conflict-stricken regions. For example, heavily conflicted areas such as Basilan, Zamboanga del Norte, Tawi-Tawi, Zamboanga Sibugay, Sulu, and Isabela remain lacking biodiversity information in the past two decades^40^.

While it is clear that war and conflict impede filling in existing knowledge gaps, the complex interplay of conflict, governmental policies, and their impact on biodiversity conservation in conflict areas constitutes a nuanced and multifaceted subject^44^. Although sociopolitical conflict can be acknowledged as offering transitory advantages to biodiversity conservation by creating no-go zones and improving vegetation recovery^45^, our evidence from the Philippines tends to be negative. Our study revealed that areas with high conflict levels, as indicated by high fatality rates, had lower tree cover and forest density. We argue that a greater variety of plants and animals may thrive in low-conflict areas, which are typically characterised by more intact ecosystems than in high-conflict areas^46–48^. Moreover, our current finding aligns with many previous studies suggesting that conflict zones often experience increased habitat destruction, ecosystem disruption, and wildlife population reduction due to the lack of statutory regulations and challenging implementation of environmental policies in high-conflict areas^10,11,49,50^. However, our results need careful interpretation, and it is crucial to explore whether the observed relationship is causal or merely correlational, considering factors like human displacement and changes in land use during conflicts^24,49,51^. If armed conflict indeed proves to be a significant driver of biodiversity loss in Mindanao, it has profound implications for conservation efforts in conflict-affected regions, necessitating collaborative strategies among policymakers, conservationists, and humanitarian organisations working in the region.

Discussions on the impact of armed conflict and violent extremism *vis-à-vis* biodiversity remain the elephant in the room, which is often neglected in biodiversity prioritisation efforts^10^, especially in the Philippines^52^. Specific connections between armed conflict and biodiversity conservation have garnered limited attention^53^. Schulte to Bühne et al. ^54^ stated that the current legal and policy frameworks regulating global biodiversity conservation do not address the challenges of conducting activities in areas affected by conflicts. They added that to incorporate conflict-sensitive protection into international policymaking, peace and scientific organisations should openly address the consequences of armed conflicts on biodiversity. Hulme^55^ and Hemptinne^56^ suggested that international environmental laws (IEL) should be reinforced and incorporate the principles of international humanitarian laws (IHL) as a tool and guide to protect the environment during and post-conflict regimes.

Another significant challenge in effectively implementing conservation initiatives in conflicted areas in the environment is the absence of baseline evidence for biodiversity status^57^. The absence of comparative data on pre- and post-conflict conditions adds complexity to our understanding of the impacts of conflict on biodiversity. Moreover, implementing biodiversity regulations and policies is particularly problematic in regions affected by conflict where maintaining law and order is difficult. Resource allocation, a key governmental responsibility, is vital for conservation initiatives; however, in conflict zones, financial resources may be redirected to address immediate security concerns, potentially affecting biodiversity conservation efforts^10,53^. Moreover, the direct and indirect impacts of sociopolitical conflicts on biodiversity, including habitat destruction and community displacement, highlight the need for mainstream post-conflict initiatives to rehabilitate and restore ecosystems^37^.

A concerted effort must be made to thoroughly document and monitor regions with notable shortfalls in biodiversity, to identify areas where gaps exist in biodiversity data, and to establish comparative monitoring assessments that employ standardised, transparent, accessible, and reproducible methods to accurately document unrecorded biodiversity. This is crucial for ensuring a comprehensive understanding of the state of biodiversity and for making informed decisions regarding conservation and management efforts^28,40^. Additionally, conservation biologists can work with local communities in conflict-affected areas to establish community-centric conservation projects. Strengthening the engagement of local communities to build awareness ensures sustainable resource management and provides economic alternatives to destructive activities. However, specific actions are needed to effectively address biodiversity shortfalls in conflict areas. Conservation biologists must integrate conflict-sensitive approaches into the planning processes. This involves considering the geopolitical context, understanding the conflict dynamics, and adapting conservation strategies accordingly. By aligning conservation efforts with local realities, we can better navigate the challenges posed by conflicts. Here, we recommend meaningful actions and considerations to bolster biodiversity conservation efforts in areas affected by sociopolitical conflict during the post-conflict period (Box 1).

In conclusion, our research sheds light on the complex and multifaceted relationship between sociopolitical conflict and biodiversity knowledge shortfalls, which restricts our understanding of biodiversity patterns in areas affected by war and conflict. Additionally, conflict zones are difficult to explore, leading to a lack of dependable biodiversity information, such as current threats and their influence on species and ecosystems. Moreover, our proposed actions extend beyond Mindanao and offer applicability to other regions that face comparable challenges. We emphasise that there is no single silver bullet that resolves the challenges brought about by sociopolitical conflicts with biodiversity. Conservation efforts in conflict zones require a multidimensional approach that addresses both ecological and sociopolitical aspects of the situation. Governmental efforts should focus on conflict prevention, post-conflict environmental restoration, initiatives to strengthen environmental governance, and engaging local communities to ensure the sustainable management of natural resources. Appropriate policies must be implemented to create a supportive environment for long-term biodiversity conservation efforts. Advocating policies and frameworks, such as the recent Kunming-Montreal Global Biodiversity Framework^2^, to bolster biodiversity conservation in conflict areas is crucial in addressing the inadequacies in these regions while simultaneously ensuring balanced benefits from nature.

Ensuring the security of conservation biologists and personnel, including park rangers, is paramount for implementing policies. Therefore, it is necessary to integrate conservation efforts into broader national security strategies^10^. Future studies should examine the extent of conflict that is identified as off-limits for researchers and conservationists. Additionally, post-conflict reconstruction and peacebuilding initiatives can offer opportunities to incorporate sustainable development and conservation measures, facilitating the restoration and protection of biodiversity in regions affected by conflict^37,53^, especially in countries with developing economies, where the overlap between conflict and biodiversity is particularly high.

Box 1 Recommended conservation strategies in conflict areas

1. *Establishment of a regional biodiversity database*: While large-scale biodiversity databases, such as the Global Biodiversity Information Facility (GBIF), are available and accessible, it is important to recognise that collecting data in the Philippines and in areas near conflict zones remains challenging. Additionally, valuable local studies may not be adequately organised and may not be readily available in biodiversity repositories (for example^40,64^). A collaborative and Findable, Accessible, Interoperable and Reproducible (FAIR) biodiversity database would be a valuable tool for harmonised conservation planning and assessment of biodiversity status on the ground^30^.
2. *Crisis mapping and data analysis*: The use of crisis mapping and data analysis tools helps scientists assess the immediate and long-term impacts of conflicts on biodiversity. This data-driven approach, based on harmonised data (see #1), aids in identifying priority areas for intervention and enables rapid response to emerging threats.
3. *Use of eDNA and barcoding technologies to survey biodiversity*: In conflict-prone areas, where it may be risky to directly observe wildlife, environmental DNA (eDNA) and DNA barcoding offer a non-intrusive way to study and monitor biodiversity^65^, even in challenging and high-conflict areas. These novel techniques can provide valuable data with or without less direct contact with the species or disturbing the environment for an extended period, which reduces exposure to threats in high-conflict areas.
4. *Biodiversity monitoring using remote-sensing technologies*: Conservation biologists can use satellite technology to monitor changes in land use and vegetation cover in conflict zones^66^ (as demonstrated in this study). This allows for the assessment of the impact of war and conflict on biodiversity, identifying areas of concern, and developing targeted spatial conservation strategies^67^.
5. *Applications of conservation drones, bioacoustics, and camera traps for surveillance*: Novel technologies, such as unmanned vehicles (i.e. drones) equipped with cameras and sensors, remote bioacoustics, and camera traps, can monitor wildlife populations and detect illegal activities in conflict zones, such as poaching and habitat destruction, in conflict zones^68^. This technology enables real-time or consolidated data collection without putting conservationists at risk (see the sections “Discussion” and “Methods”). However, the application of these technologies requires careful consideration before implementation in conflicted areas^69^.
6. *Establishment of wildlife corridors*: Conservation biologists can establish wildlife corridors connecting fragmented habitats in conflict-affected regions during the postwar period. These corridors could help maintain genetic diversity and enable species to migrate, adapt, and survive despite conflicting challenges and environmental vulnerabilities.
7. *Creation of ‘Peace Parks’*: Peace parks are transboundary conservation areas that span regions or territories affected by conflict^70^. These initiatives involve cooperation between neighbouring territories to protect shared biodiversity, promote regional stability, and encourage collaboration among communities rather than conflict.
8. *Mainstream adaptive strategies for conflict context*. Recognising the unique challenges and risks associated with conducting biodiversity studies within a conflict zone. Adapt conservation strategies that account for security considerations, access limitations, and flexibility. Develop contingency plans and implement risk management strategies to ensure the safety and well-being of personnel and local communities involved in conservation efforts.
9. *Partnership with the military for management and enforcement*: Conservation organisations and academic institutions may collaborate with governments, military forces, and international agencies to enhance the management and enforcement of protected areas in conflict zones. This involves deploying trained and experienced personnel to train the military, employing technology for biodiversity surveillance, and implementing strategies to prevent illegal activities (see #5). Additionally, military activity may be beneficial under certain conditions, such as when an exclusion zone is created, which facilitates the recovery of terrestrial and aquatic habitats^19^. This inadvertently safeguards wildlife and creates makeshift protected areas. However, reflecting on militarised actions and interventions in conservation zones is of utmost importance. Neglecting the balance between conservation and security may significantly increase the inclination to resort to violence, resulting in counterproductive and unjust consequences for both people and wildlife^21^.
10. *Promoting citizen science in post-conflict management*: Citizen science engages the public, including non-professional scientists, in biodiversity research. In areas vulnerable to war and conflict, civilians can be crucial in gathering information about their local biodiversity in the post-conflict period. By involving residents as citizen scientists and creating accessible platforms (e.g. smartphone-based biodiversity reporting^71^), valuable knowledge and access to hard-to-reach areas can be obtained remotely.

**Figure:**
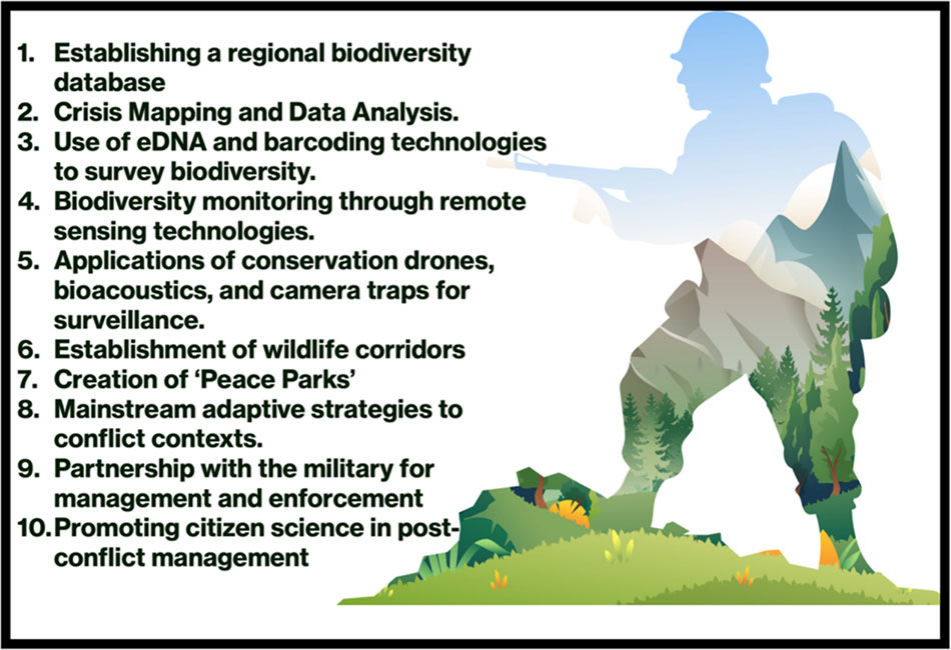
**Figure Box**. Recommended strategies to address biodiversity shortfalls within conflicted areas. Note: the image used from https://www.vecteezy.com/ under the Creative Commons Attribution-NonCommercial (BY-NC) License.

## Methods

### Comparing species richness and occurrence in conflict areas

We compared and determined the link between sociopolitical conflict and species occurrence in Mindanao. We first obtained biodiversity data from the MOBIOS^+^ database^40^ and conflict-related information for the Philippines from the UN-OCHA^36^ from 2000 to 2021. For biodiversity data, we filtered the dataset and only included biodiversity data records for insects, arachnids, fishes, amphibians, reptiles, birds, and mammals. We standardised the species occurrence data by dividing the values by the duration of the dataset and subsequently analysed the differences in species occurrence by comparing the average species richness per year between provinces with low (<10 conflicts per year) and high (>10 conflicts per year) levels of conflict using the Mann–Whitney *U* test.

We then assessed the association between the conflict events and species occurrence. To do this, we measured the distance (in m) of species occurrence records to the nearest conflict events using the ‘join attributes by nearest’ in QGIS^58^. Employing a Poisson generalised linear model, we utilised the Gamlj module within the open-source software JAMOVI 2.3.19 to predict the impacts of taxonomic groups, distance from conflict areas, and frequency of conflict events on species richness at the provincial level^59,60^. We built two GLM candidates and evaluated the best model based on the lowest values of the corrected Akaike information criterion (AICc) and Akaike weight (wAICc)^61^.

### Mapping conflicts events distribution

Assessing the impact of war and conflict on the environment is difficult in the absence of pre-war conflict data, which serves as a baseline. To provide insight into the relationship between conflict and the environment, we initially mapped and sampled conflict events to determine whether they were covered within the protected zones. We then correlated tree cover (%)^62^, tree density^47^, and forest canopy height^63^ with the number of fatalities using Pearson’s correlation coefficient (*r*). In our analysis, we used the number of fatalities per conflict event as an arbitrary measure, assuming that a higher fatality count corresponds to an increased level of conflict within a specific area.

## Data Availability

All the data used in this study are publicly available online. For consolidated Mindanao biodiversity data, the Darwin Core Formatted dataset can be accessed here 10.15468/rtedgk. Conflict records can be accessed here https://data.humdata.org/dataset/philippines-acled-conflict-data.

## References

[1] Brooks, T. M. et al. Habitat loss and extinction in the hotspots of biodiversity. *Conserv. Biol.* **16**, 909–923 (2002).

[2] Hughes, A. C. & Grumbine, R. E. The Kunming-Montreal Global Biodiversity Framework: what it does and does not do, and how to improve it. *Front. Environ. Sci* **11**, 1–12 (2023).

[3] Heberling, J. M., Miller, J. T., Noesgaard, D., Weingart, S. B. & Schigel, D. Data integration enables global biodiversity synthesis. *PNAS* **118**, e2018093118 (2021). 33526679 10.1073/pnas.2018093118 PMC8017944

[4] Borgelt, J., Dorber, M., Høiberg, M. A. & Verones, F. More than half of data deficient species predicted to be threatened by extinction. *Commun. Biol.* **5**, 1–9 (2022). 35927327 10.1038/s42003-022-03638-9 PMC9352662

[5] Mazor, T. et al. Global mismatch of policy and research on drivers of biodiversity loss. *Nat. Ecol. Evol.* **2**, 1071–1074 (2018). 29784980 10.1038/s41559-018-0563-x

[6] Hortal, J. et al. Seven shortfalls that beset large-scale knowledge of biodiversity. *Annu. Rev. Ecol. Evol. Syst.* **46**, 523–549 (2015).

[7] Moura, M. R. & Jetz, W. Shortfalls and opportunities in terrestrial vertebrate species discovery. *Nat. Ecol. Evol.* **5**, 631–639 (2021). 33753900 10.1038/s41559-021-01411-5

[8] Waldron, A. et al. Reductions in global biodiversity loss predicted from conservation spending. *Nature* **551**, 364–367 (2017). 29072294 10.1038/nature24295

[9] Robert, A. At the heart of the Vietnam War: herbicides, napalm and bulldozers against the a Lưới Mountains. *J. Alpine Res.|Rev. Géogr. Alpine*10.4000/rga.3266 (2016).

[10] Hanson, T. et al. Warfare in biodiversity hotspots. *Conserv. Biol.* **23**, 578–587 (2009). 19236450 10.1111/j.1523-1739.2009.01166.x

[11] Hanson, T. War and biodiversity conservation: the role of warfare ecology. In *Warfare Ecology* (eds Machlis, G. E., Hanson, T., Špirić, Z. & McKendry, J. E.) 125–132 (Springer Netherlands, Dordrecht, 2011).

[12] Westing, A. H. Ecological effects of military defoliation on the forests of South Vietnam. *BioScience* **21**, 893–898 (1971).

[13] Al-Mudaffar Fawzi, N., Goodwin, K. P., Mahdi, B. A. & Stevens, M. L. Effects of Mesopotamian Marsh (Iraq) desiccation on the cultural knowledge and livelihood of Marsh Arab women. *Ecosystem Health Sustain.* **2**, e01207 (2016).

[14] Clark, J. F. Foreign intervention in the civil war of the Congo Republic. *Issue* **26**, 31–36 (1998).

[15] Hotham, P. The invasion of Ukraine and its wider impact. *Fauna Flora Int.* https://www.fauna-flora.org/news/the-invasion-of-ukraine-and-its-wider-impact/ (2022).

[16] The impact of civil war on forest wildlife in West Africa: mammals in Gola Forest, Sierra Leone. *Oryx* **45**, 69–77 (2011).

[17] Saidajan, A. Effects of war on biodiversity and sustainable agricultural development in Afghanistan. *J. Dev. Sustain. Agric.* **7**, 9–13 (2012).

[18] Rawtani, D., Gupta, G., Khatri, N., Rao, P. K. & Hussain, C. M. Environmental damages due to war in Ukraine: a perspective. *Sci. Total Environ.* **850**, 157932 (2022). 35952889 10.1016/j.scitotenv.2022.157932

[19] Lawrence, M. J., Stemberger, H. L. J., Zolderdo, A. J., Struthers, D. P. & Cooke, S. J. The effects of modern war and military activities on biodiversity and the environment. *Environ. Rev.* **23**, 443–460 (2015).

[20] Duffy, R. War, by conservation. *Geoforum* **69**, 238–248 (2016).

[21] Duffy, R., John, Fa. V. S., Büscher, B. & Brockington, D. The militarization of anti-poaching: undermining long term goals? *Environ. Conserv.* **42**, 345–348 (2015).

[22] United Nations Environment Programme (UNEP). *United Nations Environment Programme (UNEP) Comments on International Law Commission (ILC) Draft Principles on Protection of the Environment in Relation to Armed Conflicts* (United Nations Environment Programme (UNEP), 2020).

[23] United Nations, U. *International Day for Preventing the Exploitation of the Environment in War and Armed Conflict* (United Nations, 2017).

[24] Dean, W. R. J., Melo, M. & Mills, M. S. L. The avifauna of Angola: richness, endemism and rarity. In *Biodiversity of Angola: Science & Conservation: A Modern Synthesis* (eds Huntley, B. J., Russo, V., Lages, F. & Ferrand, N.) 335–356 (Springer International Publishing, Cham, 2019).

[25] PhilAtlas. *Mindanao—PhilAtlas* (PhilAtlas, 2021).

[26] Asian Development Bank. *Mindanao Agro-Enterprise Development Project* (Asian Development Bank, 2023).

[27] Heaney, L. R. & Regalado, J. C. Vanishing treasures of the Philippine Rain Forest. *J. Mammal.* **82**, 246–247 (2001).

[28] Dela Cruz, K. C., Abdullah, S. S., Agduma, A. R. & Tanalgo, K. C. Early twenty-first century biodiversity data pinpoint key targets for bird and mammal conservation in Mindanao, Southern Philippines. *Biodiversity* **24**, 146–163 (2023).

[29] Ambal, R. G. R. et al. Key biodiversity areas in the Philippines: priorities for conservation. *J. Threatened Taxa* **4**, 2788–2796 (2012).

[30] Agduma, A. et al. Overview of priorities, threats, and challenges to biodiversity conservation in the Southern Philippines. *Reg. Sustain.* **4**, 1–12 (2023).

[31] Abdullah, S. S. et al. Leaping forward or crawling backward? Efforts and biases in Amphibian and Reptile research on a megadiverse faunal region in the Philippines. *Conservation* **3**, 363–378 (2023).

[32] Abubakar, C. A. Review of the Mindanao peace processes. *Inter-Asia Cult. Stud.* **5**, 450–464 (2004).

[33] Montiel, C. J., Rodil, R. B. & de Guzman, J. M. The Moro struggle and the challenge to peace building in Mindanao, Southern Philippines. In *Handbook**of Ethnic**Conflict*: *International Perspectives* (eds Landis, D. & Albert, R. D.) 71–89 (Springer US, Boston, MA, 2012).

[34] Brown, G. The long and winding road: the peace process in Mindanao, Philippines. *IBIS Discuss. Paper* **6**, 1–42 (2023).

[35] Ferrer, R. B. & Cabangbang, R. G. Non-International armed conflicts in the Philippines. *Int. Law Stud.* **88**, 263–278 (2012).

[36] UN OCHA. *Conflict Data for Philippines* (Humanitarian Data Exchange, 2023).

[37] Rodríguez, A. C. T. et al. Answering the right questions. Addressing biodiversity conservation in post-conflict Colombia. *Environ. Sci. Policy* **104**, 82–87 (2020).

[38] Tanalgo, K. C. & Hughes, A. C. Bats of the Philippine Islands—a review of research directions and relevance to national-level priorities and targets. *Mammalian Biol.* **91**, 46–56 (2018).

[39] Gamalo, L. E., Sabanal, B. & Ang, A. Three decades of Philippine nonhuman primate studies: research gaps and opportunities for Philippine primatology. *Primates* **62**, 233–239 (2021). 32681352 10.1007/s10329-020-00847-w

[40] Tanalgo, K. et al. The MOBIOS+: a FAIR (Findable, Accessible, Interoperable and Reusable) database for Mindanao’s terrestrial biodiversity. *Biodivers. Data J.* **11**, e110016 (2023). 38312338 10.3897/BDJ.11.e110016 PMC10838081

[41] South, A. & Joll, C. M. From Rebels to Rulers: the challenges of transition for non-state armed groups in Mindanao and Myanmar. *Crit. Asian Stud.* **48**, 168–192 (2016).

[42] Peña, K. D. *Maguindanao Massacre: the Wound of PH Impunity That Will Never Heal*. INQUIRER.net https://newsinfo.inquirer.net/1696507/maguindanao-massacre-the-wound-of-ph-impunity-that-will-never-heal (2022).

[43] Alipala, J. *Tawi-Tawi Gov Says 2 Kidnapped Bird Watchers Moved by Abus to Sulu*. INQUIRER.net https://globalnation.inquirer.net/35925/tawi-tawi-gov-says-2-kidnapped-bird-watchers-moved-by-abus-to-sulu (2012).

[44] Young, A. The military’s responsibility for environmental protection in war and peace. *Environ. Sci. Pollut. Res. Int.* **10**, 203–204 (2003). 12943001 10.1007/BF02980232

[45] Dávalos, L. M. The San Lucas mountain range in Colombia: how much conservation is owed to the violence? *Biodivers. Conserv.* **10**, 69–78 (2001).

[46] Tao, S., Guo, Q., Li, C., Wang, Z. & Fang, J. Global patterns and determinants of forest canopy height. *Ecology* **97**, 3265–3270 (2016). 27912007 10.1002/ecy.1580

[47] Crowther, T., Glick, H. & Covey, K. *Global Tree Density Map* https://elischolar.library.yale.edu/yale_fes_data/1/ (2015).

[48] Tanalgo, K. C., Oliveira, H. F. M. & Hughes, A. C. Mapping global conservation priorities and habitat vulnerabilities for cave-dwelling bats in a changing world. *Sci. Total Environ.* **843**, 156909 (2022). 35753458 10.1016/j.scitotenv.2022.156909

[49] Bautista-Cespedes, O. V., Willemen, L., Castro-Nunez, A. & Groen, T. A. The effects of armed conflict on forest cover changes across temporal and spatial scales in the Colombian Amazon. *Reg. Environ. Change* **21**, 70 (2021).

[50] Arakwiye, B., Rogan, J. & Eastman, J. R. Thirty years of forest-cover change in Western Rwanda during periods of wars and environmental policy shifts. *Reg. Environ. Change* **21**, 27 (2021).

[51] Sánchez-Cuervo, A. M. & Aide, T. M. Consequences of the armed conflict, forced human displacement, and land abandonment on forest cover change in Colombia: a multi-scaled analysis. *Ecosystems* **16**, 1052–1070 (2013).

[52] Agduma, A. R. et al. Diversity of vascular plant species in an agroforest: the case of a rubber (*Hevea brasiliensis*) plantation in Makilala, North Cotabato. *Philippine J. Crop Sci.* **36**, 57–64 (2011).

[53] International Union for Conservation of Nature and Natural Resources. *Conflict and Conservation* (International Union for Conservation of Nature and Natural Resources, 2021).

[54] Schulte to Bühne, H., Pettorelli, N. & Hoffmann, M. The policy consequences of defining rewilding. *Ambio* **51**, 93–102 (2022). 33983560 10.1007/s13280-021-01560-8 PMC8651963

[55] Hulme, K. Using International Environmental Law to enhance biodiversity and nature conservation during armed conflict. *J. Int. Criminal Justice* **20**, 1155–1190 (2022).

[56] de Hemptinne, J. Increasing the safeguarding of protected areas threatened by warfare through International Environmental Law. *Int. Rev. Red Cross* **105**, 1392–1411 (2023).

[57] Weir, D., McQuillan, D. & Francis, R. A. Civilian science: the potential of participatory environmental monitoring in areas affected by armed conflicts. *Environ. Monit. Assess.* **191**, 618 (2019). 31493019 10.1007/s10661-019-7773-9 PMC6731190

[58] QGIS Development Team. *QGIS Geographic Information System. Open Source Geospatial Foundation Project* (QGIS Development Team, 2022).

[59] Gallucci, M. *GAMLj: General Analyses for the Linear Model in Jamovi* [Computer Software] (2019).

[60] The Jamovi Project. *Jamovi (Version 2.3.22) [Computer Software]* (The Jamovi Project, 2023).

[61] Burnham, K. P. & Anderson, D. R. Multimodel inference: understanding AIC and BIC in model selection. *Sociol. Methods Res.* **33**, 261–304 (2004).

[62] Hansen, A. J. et al. Global change in forests: responses of species, communities, and biomes. *BioScience* **51**, 765 (2001).

[63] Potapov, P. et al. Mapping global forest canopy height through integration of GEDI and Landsat data. *Remote Sens. Environ.* **253**, 112165 (2021).

[64] Tanalgo, K. C., Achondo, M. J. M. M. & Hughes, A. C. Small things matter: the value of rapid biodiversity surveys to understanding local bird diversity patterns in Southcentral Mindanao, Philippines. *Trop. Conserv. Sci.* **12**, 1940082919869482 (2019).

[65] Beng, K. C. & Corlett, R. T. Applications of environmental DNA (eDNA) in ecology and conservation: opportunities, challenges and prospects. *Biodivers. Conserv.* **29**, 2089–2121 (2020).

[66] Kaplan, G., Rashid, T., Gasparovic, M., Pietrelli, A. & Ferrara, V. Monitoring war-generated environmental security using remote sensing: a review. *Land Degrad. Dev.* **33**, 1513–1526 (2022).

[67] Hanson, T. Biodiversity conservation and armed conflict: a warfare ecology perspective. *Ann. N. Y. Acad. Sci.* **1429**, 50–65 (2018). 29683517 10.1111/nyas.13689

[68] Bergenas, J., Stohl, R. & Georgieff, A. The other side of drones: saving wildlife in Africa and managing global crime. *Confl. Trends* **2013**, 3–9 (2013).

[69] Dasgupta, D. When wildlife surveillance tech ‘watches’ people. *Mongabay Environ. News* https://news.mongabay.com/2023/07/when-wildlife-surveillance-tech-watches-people/ (2023).

[70] McNeely, J. A. Conserving forest biodiversity in times of violent conflict. *Oryx* **37**, 142–152 (2003).

[71] Andrachuk, M., Marschke, M., Hings, C. & Armitage, D. Smartphone technologies supporting community-based environmental monitoring and implementation: a systematic scoping review. *Biol. Conserv.* **237**, 430–442 (2019).

